# High-throughput screening identifies histone deacetylase inhibitors that modulate GTF2I expression in 7q11.23 microduplication autism spectrum disorder patient-derived cortical neurons

**DOI:** 10.1186/s13229-020-00387-6

**Published:** 2020-11-19

**Authors:** Francesca Cavallo, Flavia Troglio, Giovanni Fagà, Daniele Fancelli, Reinald Shyti, Sebastiano Trattaro, Matteo Zanella, Giuseppe D’Agostino, James M. Hughes, Maria Rosaria Cera, Maurizio Pasi, Michele Gabriele, Maddalena Lazzarin, Marija Mihailovich, Frank Kooy, Alessandro Rosa, Ciro Mercurio, Mario Varasi, Giuseppe Testa

**Affiliations:** 1 Department of Oncology and Hemato-Oncology, University of Milan, c/o High Definition Disease Modelling Lab: Stem Cell and Organoid Epigenetics, IEO, European Institute of Oncology IRCCS, Via Adamello 16, 20139 Milan, Italy; 2 High Definition Disease Modelling Lab: Stem Cell and Organoid Epigenetics, IEO, European Institute of Oncology IRCCS, Via Adamello 16, 20139 Milan, Italy; 3 Department of Experimental Oncology, European Institute of Oncology IRCCS, Via Adamello 16, 20139 Milan, Italy; 4 Present Address: IFOM, the FIRC Institute of Molecular Oncology, Via Adamello 16, 20139 Milan, Italy; 5 Department of Biology and Biotechnology Charles Darwin, Sapienza University of Rome, P.le A. Moro 5, 00185 Rome, Italy; 6 Center for Life Nano Science, Istituto Italiano Di Tecnologia, Viale Regina Elena 291, 00161 Rome, Italy; 7 Department of Oncology and Hemato-Oncology, University of Milan, Via Santa Sofia 9, 20122 Milan, Italy; 8 Human Technopole, Via Cristina Belgioioso, 171, 20157 Milan, Italy; 9 Department of Medical Genetics, University of Antwerp, Antwerp, Belgium; 10 Present Address: Evotec SE, Hamburg, Germany; 11 Present Address: Lee Kong Chian School of Medicine, Nanyang Technological University, Singapore, Singapore; 12 Present Address: FPO - IRCCS, Candiolo Cancer Institute, SP 142 Km 3.95, 10060 Candiolo, TO Italy; 13 Present Address: Department of Biological Engineering, Massachusetts Institute of Technology, Cambridge, USA

**Keywords:** Autism spectrum disorder, 7q11.23 duplication syndrome, Intellectual disability, High-throughput screening, HDAC inhibitors, Induced pluripotent stem cells, Neurons, GTF2I

## Abstract

**Background:**

Autism spectrum disorder (ASD) is a highly prevalent neurodevelopmental condition affecting almost 1% of children, and represents a major unmet medical need with no effective drug treatment available. Duplication at 7q11.23 (7Dup), encompassing 26–28 genes, is one of the best characterized ASD-causing copy number variations and offers unique translational opportunities, because the hemideletion of the same interval causes Williams–Beuren syndrome (WBS), a condition defined by hypersociability and language strengths, thereby providing a unique reference to validate treatments for the ASD symptoms. In the above-indicated interval at 7q11.23, defined as WBS critical region, several genes, such as *GTF2I*, *BAZ1B*, *CLIP2* and *EIF4H*, emerged as critical for their role in the pathogenesis of WBS and 7Dup both from mouse models and human studies.

**Methods:**

We performed a high-throughput screening of 1478 compounds, including central nervous system agents, epigenetic modulators and experimental substances, on patient-derived cortical glutamatergic neurons differentiated from our cohort of induced pluripotent stem cell lines (iPSCs), monitoring the transcriptional modulation of WBS interval genes, with a special focus on *GTF2I*, in light of its overriding pathogenic role. The hits identified were validated by measuring gene expression by qRT-PCR and the results were confirmed by western blotting.

**Results:**

We identified and selected three histone deacetylase inhibitors (HDACi) that decreased the abnormal expression level of GTF2I in 7Dup cortical glutamatergic neurons differentiated from four genetically different iPSC lines. We confirmed this effect also at the protein level.

**Limitations:**

In this study, we did not address the molecular mechanisms whereby HDAC inhibitors act on GTF2I. The lead compounds identified will now need to be advanced to further testing in additional models, including patient-derived brain organoids and mouse models recapitulating the gene imbalances of the 7q11.23 microduplication, in order to validate their efficacy in rescuing phenotypes across multiple functional layers within a translational pipeline towards clinical use.

**Conclusions:**

These results represent a unique opportunity for the development of a specific class of compounds for treating 7Dup and other forms of intellectual disability and autism.

## Background

Autism spectrum disorder (ASD) comprises a highly prevalent group of neurodevelopmental disorders (NDD) affecting almost 1% of children. Children diagnosed with ASD exhibit impairments in language and social interaction coupled to stereotyped behaviors and, in many cases, the co-occurrence of varying degrees of intellectual disability (ID) [1]. Due to its extremely high prevalence and the lack of effective therapies, ASD represents a major unmet medical need. Despite the phenotypic convergence of its core symptoms (modulated over an ample range of expressivity, whence the term spectrum), ASD is phenotypically and genetically highly heterogeneous with over 400 identified causal genetic alterations reinforcing the view of ASD as a collection of rare genetic conditions [2]. The presence of similar core symptoms across the genetic spectrum of ASD suggests that few paradigmatic syndromes might make the understanding of ASD causes and therapeutic interventions feasible.

**Fig. 1:**
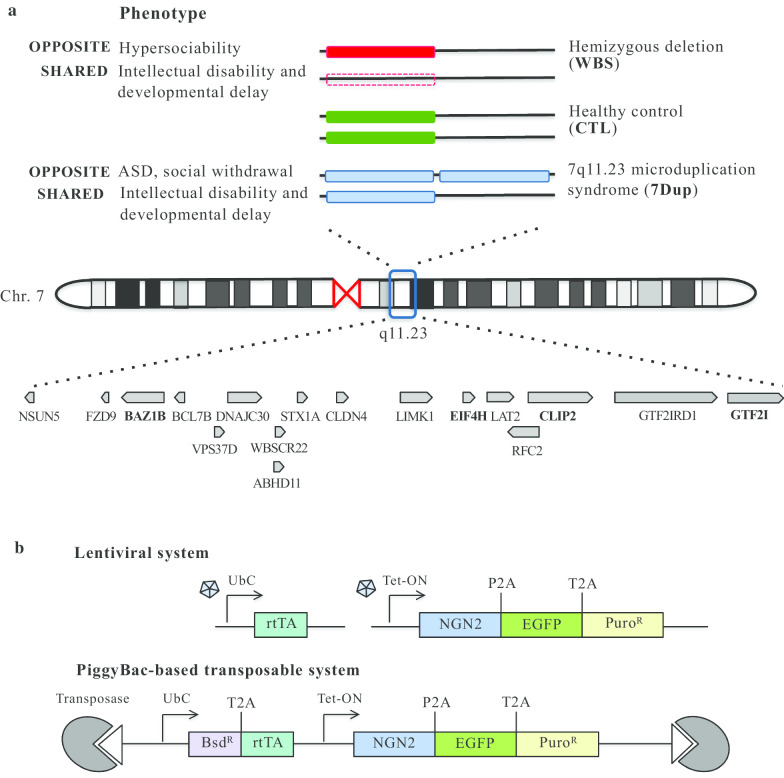
Symmetric copy number variations at 7q11.23. **a** Schematic representation of genotype–phenotype correlation in WBS and 7Dup patients, compared to healthy control (CTL), indicating opposite and shared phenotypes. Genomic organization of WBS region with the 17 genes that are significantly expressed in neurons, in bold the four genes selected for their critical role in the pathogenesis of both WBS and 7Dup. **b** Lentiviral vector (top) and PiggyBac-based construct (bottom) used to induce iPSC differentiation into cortical neurons. Ubc: human Ubiquitin constitutive promoter, rtTA: TET transactivator promoter gene, NGN2: Neurogenin2 gene, Puro^R^: Puromycin resistance gene, Bsd^R^: Blasticidin resistance gene, white triangles represent terminal repeats of the transposon

As a matter of fact, duplication of a segment of chromosome 7 at 7q11.23 comprising 26–28 genes, one of the best-characterized copy number variations (CNVs) underlying ASD (7Dup) [3, 4], might yield invaluable insights into ASD pathophysiology, also because it is symmetrically opposite to the hemideletion of the same interval that causes Williams–Beuren Syndrome (WBS), a multisystemic disease including hypersociability and selectively spared verbal abilities despite their mild to moderate ID and a severely compromised visual–spatial processing and planning [5]. Almost all WBS patients have mild to moderate ID, while only a minority of 7Dup patients show ID. Moreover, both syndromes are characterized by anxiety and attention deficit hyperactivity disorder (ADHD). 7Dup patients show a range of ASD traits, especially in terms of varying degrees of language impairments and social restriction (Fig. 1a). The combination of symmetrically opposite CNVs resulting into symmetrically opposite behavioral phenotypes offers unique opportunities to dissect the dosage-vulnerable circuits that affect language competence and sociability.

Consequently, compounds that modulate gene dosage alterations may provide therapeutic options into ASD pathophysiology that so far has been notoriously difficult. To date, several disease modeling studies have demonstrated that the use of different induced pluripotent stem cells (iPSCs)-derived cell types in different disease-relevant conditions is suitable for high-throughput screening (HTS), confirming the high potential of iPSCs and their differentiated derivatives in pharmacological research [6–9]. The process from basic research to bedside is very long, expensive, and poses a number of risks and difficulties along the way, with the result that the number of new potential therapeutic compounds that actually become drugs is very low. The process of drug repositioning makes the drug discovery process much shorter because the initial phases of drug discovery have already been performed. Therefore, this represents a unique alternative tool for the unmet medical need related to many genetic diseases, including neurodevelopmental disorders [10, 11].

Among the different classes of existing drugs, histone deacetylase inhibitors (HDACi) are an interesting category of therapeutics with potential as anticancer drugs [12]. There is a vast literature demonstrating the involvement of HDACs in suppressing critical genes in different types of cancer, including brain tumors [13–15]. Interestingly, HDACi are now being considered as potential therapeutics also for neuropsychiatric disorders [16, 17].

Among the genes of the 7q11.23 region, general transcription factor II-I (GTF2I) has key relevance: it mediates signal-dependent transcription and plays a prominent role in various signaling pathways [18]. Most importantly, convergent evidence has implicated GTF2I as a major mediator of the cognitive-behavioral alterations in 7Dup [19–21]. Using iPSCs from 7Dup patients, we discovered that GTF2I is responsible for a large part of transcriptional dysregulation, evident at the pluripotent state, which is amplified upon differentiation into neural progenitors. Specifically, GTF2I recruits lysine demethylase 1 (LSD1) to repress transcription of critical neuronal genes, an effect that is rescued by inhibition of LSD1 [22], a potential target for therapeutic intervention.

On the basis of these convergent lines of evidence, the identification, in patient-derived neurons, of compounds that restore normal expression of genes from CNV causative of ASD represents a promising upstream strategy to develop novel therapies for ASD.

In this work, we set out to identify compounds that can restore the expression levels of key genes from the WBSCR, i.e., increasing or decreasing their expression in, respectively, WBS or 7Dup neurons. For the above reasons, we selected as targets *GTF2I* along with three additional genes within the 7q11.23 region, namely *BAZ1B, CLIP2* and *EIF4H*, that emerged as critical for their role in the pathogenesis of WBS and 7Dup [23]. In particular, *BAZ1B* is a chromatin remodeler that is involved in maintenance and migration of neural crest cells playing an important role in the evolution of modern human faces and thus being a prime candidate to study disease-associated craniofacial alterations [24, 25]; *CLIP2* is a microtubule-binding protein abundantly expressed in neurons whose haploinsufficiency might contribute to the cerebellar and hippocampal dysfunctions observed in the WBS [26]; *EIF4H* is a translation initiation factor mediating protein synthesis that might be involved in growth retardation in *EIF4H* knockout mice [27, 28].

## Methods

### Human samples

Participation by patients and their relatives in the studies [22, 25], that led to the establishment of iPSC lines, were approved by the respective ethics committees following the informed consent and ethics review procedures in place. The fibroblast biological samples from which we derived the iPSC lines harboring 7q11.23 CNV, along with the controls, are archived in the Genetic and Genomic Disorders Biobank (GDBank), which is part of the Telethon Network of Genetic Biobanks (TNGB). https://www.telethon.it/en/scientists/biobanks. The primary fibroblasts samples were collected from the following sources:

The Genetic and Genomic Disorders Biobank (GDBank) (Dr. Giuseppe Merla, Casa Sollievo della Sofferenza, San Giovanni Rotondo (Italy) for samples Ctl01C (Female), WBS01C (Male), WBS02C (F), Dup02K (F), Dup03B (M). Dr. Paolo Prontera, University of Perugia (Azienda Ospedaliera–Universitaria “Santa Maria della Misericordia”, Perugia, Italy) for line Dup01G (M). Dr. Frank Kooy, University of Antwerp, Antwerp (Belgium) for line Dup04A (M). The Wellcome Trust Sanger Institute, Cambridge (UK) provided upon purchase the iPSC line Ctl08A (M).

A SNPs profiling was performed on iPSC lines using the Illumina GSA beadchip GSA MD v1 kit (Illumina GSA Arrays “Infinium iSelect 24 × 1 HTS Custom Beadchip Kit”) by The Human Genomics Facility of the Genetic Laboratory of the Department of Internal Medicine at Erasmus MC (Rotterdam). We regularly perform genomic quality control of cell lines used by short tandem repeat (STR)-based approach (Additional file 1: Table S1).

Fibroblasts were reprogrammed using the mRNA Reprogramming kit (Stemgent) or with the microRNA Booster kit, as previously described [22, 25].

### iPSCs lentiviral infection and PiggyBac system

Patient-derived iPSC lines were infected with an activator lentivirus, containing the reverse tetracycline transactivator (rtTA) constitutively expressed under the control of the UbC promoter, and an effector lentivirus, containing an NGN2-P2A-EGFP-T2A-Puro cDNA under the control of the tetracycline responsive element [29] (Fig. 1b, top). Infected iPSCs were sorted as single cells in 96-well plates, selected based on the round morphology of colonies and gradually expanded. Selected lines were then induced for one day adding doxycycline to the medium. GFP-positive lines were then selected and expanded, further being stabilized and characterized. Through this system, we generated the iPSC monoclonal lines WBS01CN3 (WBS) and DUP01GN4 (7Dup).

To establish a robust and rapid neuronal differentiation method, we utilized a direct conversion technology. Mouse Ngn2 cDNA, under tetracycline-inducible promoter (tetO), was transfected into iPSCs by a newly developed enhanced PiggyBac (ePB) transposon system [9, 30, 31] (Fig. 1b, bottom). 4 × 10^5^ iPSCs, for each line, were electroporated with 2.25 μg of the ePB construct carrying the inducible Neurogenin-2 (NGN2) overexpression cassette and 250 ng of the plasmid encoding transposase for the genomic integration of the inducible cassette. Electroporations were performed using the Neon Transfection System (MPK10096, Thermo Fisher Scientific). iPSCs were selected using blasticidin 5 μg/ml (R21001, Gibco) for five days and stable iPSC lines were stocked. Through the ePB system, we generated the following polyclonal lines: Ctl01C, Ctl08A (CTL): WBS01C, WBS02C (WBS); Dup03B, Dup04A, Dup01G, Dup02K (7Dup).

### NGN2 differentiation into cortical glutamatergic neurons

In order to obtain cortical glutamatergic neurons (iNs), on day -1 iPSCs were dissociated with Accutase (GIBCO, Thermo Fisher Scientific) and seeded in plates coated with 2.5% (v/v) Matrigel (Corning) in mTeSR™ supplemented with ROCK inhibitor (STEMCELL Technologies). iPSCs were then cultured in MEM1, composed by DMEM/F12 1:1 (Euroclone/Gibco) supplemented with NEAA 1%, N2 1%, BDNF 10 ng/ml, NT-3 10 ng/ml, Laminin 0.2 μg/ml and 2 μg/ml doxycycline hydrochloride, from day 0 to day 1. On day 1, puromycin 1 μg/ml was added to MEM1 and, on day 2, the medium was changed with NBM Plus, composed by Neurobasal Plus (Thermo Fisher Scientific) supplemented with 50 × B27 Plus supplement (GIBCO, Thermo Fisher Scientific), Glutamax 0.25% (Thermo Fisher Scientific) and 2 μg/ml doxycycline hydrochloride. On day 7, differentiated neuronal cells were dissociated with Accutase and seeded into poly-d-lysine-coated 96-well plates (Corning) at a density of 20.000 cells/well in NBM Plus; culture medium was then changed 50% once a week until day 28.

### Immunocytochemistry

Neurons were fixed in 4% paraformaldehyde in PBS for 15 min at room temperature immediately after removal of culture medium, and pipetting was done slowly to prevent dislodging cells from coverslips. The cells were then washed three times for 5 min with PBS, permeabilized with 0.1% Triton X-100 in PBS for 15 min, and blocked in 5% donkey serum in PBS for 30 min. After blocking, the cells were incubated with primary antibodies diluted in blocking solution overnight at + 4 °C. The cells were washed three times with PBS for 5 min and incubated with secondary antibodies at room temperature for 1 h. Nuclei were then stained with DAPI solution at room temperature for 10 min. Coverslips were rinsed in sterile water and mounted on a glass slide with 7–8 μl of Mowiol mounting medium.

### Cellomics

Neurons in 96-well plates were fixed in 4% paraformaldehyde in PBS, permeabilized with 0.1% Triton X-100 and then counterstained with DAPI to enable autofocusing of the automated Thermo Scientific ArrayScan VTI High-content screening microscope (Cellomics). Cell counting of validated objects was done in the DAPI channel and in the GFP channel.

### Automation protocol

All liquid handling was done in an automated manner by a TECAN Freedom EVO automated platform under control of EVOware® software. The TECAN has been programmed to prepare up to 20 96-well plates in a single run, which would produce 1080 individual datapoints. The modular robotic scripts were designed as building blocks for users with minimal automation programming experience to assemble an automated process from cells preparation to sample analysis. We prepared scripts for compound treatment, RNA and cDNA dilution (and predilution if necessary), reagent addition (Cells-to-CT, RT), and samples re-positioning in pre-spotted 384-well plates. Each module contained user-friendly interfaces for inputs of assay variables, such as volumes, dilution factors and plate maps. The liquid-handling robot used in this work is a Tecan Freedom EVO-2 150 liquid handling unit equipped with a 96-well head-adapter with filter tips; the pipetting volume range was from 10 to 1000 μl. The Freedom EVO worktable was loaded with three solution reservoir carriers (1 × Trough 100 ml, 3 Pos. and 2 × Trough 25 ml, 3 Pos.), two 96-well plate carriers (96-well, 6 Pos.), and one 384-well plate carrier (96-well, 3 Pos.).

### Quantitative RT-PCR

A custom TaqMan Cells-to-CT™ kit (Invitrogen AM1729) was used to extract the RNA and perform reverse transcription to obtain cDNA, according to the manufacturer’s instructions. After media aspiration, 30 μl of 2 × lysis solution, with diluted DNaseI, were added to 30 μl of the remaining buffer in each well; then the plate was incubated for 5 min at room temperature. Subsequently Stop solution (3 μl) was added and the solution was incubated at room temperature for 2 min. Then, 30 μl of lysates was transferred to a new PCR plate with 40 μL of reverse transcription enzyme mix previously added to each well. The thermal cycling conditions were: 60 min at 42 °C, and 5 min at 85 °C. cDNA was diluted with 50 μl of water and then a 5 μl aliquot of each cDNA reaction was added to 5 μl of each TaqMan master mix reaction into pre-spotted custom 384-well plates. A QuantStudio 6 Flex Real-Time PCR system (Applied Biosystems) was utilized to determine the Ct values. Relative mRNA expression levels were normalized to housekeeping genes and analyzed through the comparative delta-delta Ct method using the QBase Biogazelle software.

### Hit selection

We used a strategy based on fold-difference analysis of target genes, comparing compound- to DMSO control-treated wells. Hits were defined as more than twofold increase or less than 0.5-fold decrease in at least three out of four genes, or in at least *GTF2I*. Thirty-five compounds fulfilled the first criteria and 36 compounds the second one in the primary screening.

### Antibodies

The following antibodies were used for Western Blot analysis: pAb anti-GTF2I 1:1000 (A301-330A, Bethyl Laboratories), pAb anti-GAPDH 1:5000 (ABS16, Merck Millipore) and secondary antibody horseradish peroxidase-conjugated donkey anti-rabbit (Pierce). The following antibodies were used for immunocytochemistry analyses: NeuN 1:500 (MABN140, Sigma-Aldrich), TUBB3 1:1000 (PRB-435P, BioLegend), MAP2 1:500 (M9942, Sigma-Aldrich), vGlut1 1:1000 (135303, Synaptic Systems), SATB2 1:200 (ab51502, Abcam), Synapsin 1/2 1:1000 (106004, Synaptic Systems).

### Chemicals

Epigenetics compound library: Selleckchem Cat. N° L1900; Bioactive compound library: Food and Drug Administration (FDA) approved and clinical compounds selected from the Library of Pharmacologically Active Compounds (LOPAC, Sigma) and the Spectrum Collection (MicroSource Inc). Both in the primary screening and in validation experiments, a single dose of each compound was added to neurons at the final concentration of 10 μM in 0.1% DMSO for 48 h.

### Protein extraction and immunoblotting

Proteins were extracted from iNs grown in 10 cm or six-well plates by washing the cells with ice-cold PBS, followed by immersion in lysis buffer (25 mM Hepes pH 7.5, 300 mM NaCl, 10% glycerol, 1% NP-40) supplemented with cOmplete™ protease inhibitor cocktail (Sigma). Lysates were sonicated using the Bioruptor Sonication System (UCD200) for three cycles of 30 s with 60-s breaks at high power and then centrifuged at 13,000*g* for 15 min. Protein quantification was performed using the Bradford protein assay (Bio-Rad) following the manufacturer's instructions. Protein extracts (10–20 μg per sample) were run on a precast NuPAGE 4–12% Bis–Tris Gel (NP0335BOX, Life Technologies), transferred to a nitrocellulose membrane and blocked in TBST (50 mM Tris, pH 7.5, 150 mM NaCl and 0.1% Tween-20) and 5% milk at room temperature for 1 h. Primary and secondary antibodies were diluted in TBST and 5% milk. The immunoreactive bands were detected by ECL (GE Healthcare) and imaged with a ChemiDoc XRS system (Bio-Rad Laboratories). Densitometric analysis was performed using the ImageLab 4.1 Software (Bio-Rad Laboratories).

### Data analysis

Qbase + software version 3.0 (Biogazelle, Zwijnaarde, Belgium) was used to analyze the variability of the genes tested and to determine the hit compounds. The geometric mean of the cycle threshold value of the endogenous control genes GAPDH, SRSF9 and RPS18 was used to normalize the data, and the DMSO-treated samples were used as calibrator.

### Statistical analysis

Statistical analyses were performed using PRISM (GraphPad, version 6.0). Results are expressed as means ± SD or means ± SE. Statistical significance was determined according Holm–Sidak-corrected *t* test, considering each iPSC line as biological replicate (n) or according to a one way ANOVA test as indicated in figure legends. Dunnett's multiple comparison test was used to determine the level of significance. Asterisks indicate statistical significance (*P < 0.05, **P < 0.005, ***P < 0.0005, ****P < 0.0001).

## Results

### NGN2-driven neurogenesis retains the defining transcriptional imbalances of 7q11.23 CNV

With the goal of identifying compounds capable of restoring the physiological expression levels of the four aforementioned genes from the WBSCR, we set out to establish HTS-proof conditions for the differentiation and maintenance of patient-derived cortical neurons, starting off with the NGN2-driven system of iPSC differentiation [29] and adapting it to HTS as follows.

First, we reasoned that, in a HTS setting inherently prone to fluctuations in numerous technical variables, the use of lines with a fixed number of integrations would help reduce the confounding variables intrinsic to the differentiation of polyclonal batches with an unchecked diversity of copy number integrations of the NGN2 transgene. Thus, we used an NGN2 expressing lentivirus to generate a stable monoclonal iPSC line originally reprogrammed from a patient harboring the WBS deletion (hereafter WBS01CN3 line).

**Fig. 2:**
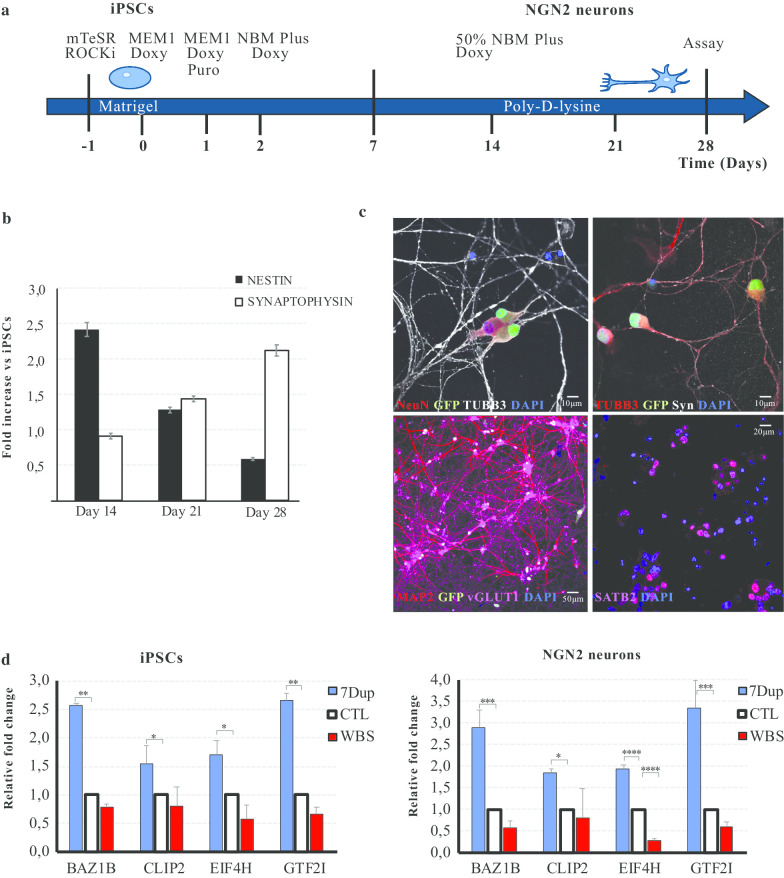
NGN2-mediated conversion of iPSCs to iNs. **a** Timeline of differentiation protocol for iPSC-derived cortical neurons. ROCKi: ROCK inhibitor; Doxy: doxycycline; Puro: puromycin. **b** RT-qPCR analysis of Nestin and Synaptophysin mRNA expression (mean ± SE) in NGN2 neurons (WBS01CN3 line) at 2, 3, and 4 weeks of differentiation. The expression level is normalized against GAPDH, and further standardized to iPSCs levels. **c** Day 28 WBS01CN3 neurons express mature excitatory cortical neuron markers: NeuN, TUBB3, Synapsin 1/2, MAP2, VGLUT1 and SATB2. **d** mRNA levels of genes in the WBS region, BAZ1B, CLIP2, EIF4H and GTF2I (mean ± SD), in iPSC lines (left) and in NGN2-induced neurons (right) in the three genotypes (WBS, CTL, 7Dup) (*n* = 2). CTL: Ctl01C, Ctl08A; WBS: WBS01CN3, WBS02C; 7Dup: DUP01GN4, Dup02K. Relative expression was measured by RT-qPCR, to GAPDH and results were arbitrarily normalized to mRNA levels of CTL (asterisks indicate statistical significance according to a one-way ANOVA test: *P < 0.05, **P < 0.005, ***P < 0.0005, ****P < 0.0001)

Second, since the original NGN2-driven protocol [29] relied on astrocytes to support neuronal growth but their presence would have interfered with gene expression analysis in neurons, we used a new formulation medium (NBM Plus) that allowed to replace astrocytes and minimize media changes, thus also reducing the automation complexity of the HTS. Third, we adapted the differentiation protocol to a HTS platform by first seeding the iPSCs and culturing them in large batches on Matrigel-coated 15 cm dishes and then detaching them for seeding on poly-d-lysine coated 96-well plates (Fig. 2a). We validated the robustness of this protocol by both immunocytochemistry and RT-qPCR. Forced NGN2 expression converted iPSCs into mature neuronal morphology in 28 days with a rapid decline of the neural progenitor marker Nestin and an increase in the expression of the synaptic marker Synaptophysin (Fig. 2b). iPSC-derived NGN2-induced neurons (iNs) express glutamatergic markers like vGLUT1, cortical markers such as SATB2 and the expected combination of both early neuron markers like TUBB3 and mature neuron markers as MAP2, NeuN and the synaptic marker Synapsin 1/2 (Fig. 2c).

We also analyzed the expression levels of *SATB2*, *MAP2* and *SYN1* in both WBS and 7Dup iNs compared to healthy control (CTL) iNs, using Syntaxin1A (*STX1A*), a WBSCR gene, as internal control of symmetrical dosage imbalance. While *SATB2* and *SYN1* show an increased expression in both 7Dup and WBS iNs compared to CTL, *MAP2* does not show 7q11.23 dosage-dependent alterations in expression levels (Additional file 2: Fig. S1A). Moreover, as a baseline evaluation of HTS-relevant neuronal morphology, we performed a morphometric Sholl analysis of dendrites in WBS, 7Dup and CTL iNs, plotting the number of intersections with circles centered on the soma against the distance from the cell body. Detailed analysis of neurons revealed unaltered complexity for both basal and apical dendrites across the three genotypes (Additional file 2: Fig. S1B, C), unlike what was previously reported [32], likely reflecting a differential expressivity of 7q11.23 CNV, in terms of morphological readout, dependent on different protocols of neuronal differentiation.

Finally, we confirmed that the transcriptional levels of *BAZ1B, CLIP2, EIF4H* and *GTF2I* mirrored the symmetrical gene dosage in both WBS and 7Dup iNs compared to CTL, confirming that the gene dosage imbalance is maintained and amplified upon cortical neuronal differentiation (Fig. 2d) and hence that it represents a rational target for a mechanistically based therapeutic intervention.

### Establishment of an in vitro platform for drug screening

**Fig. 3:**
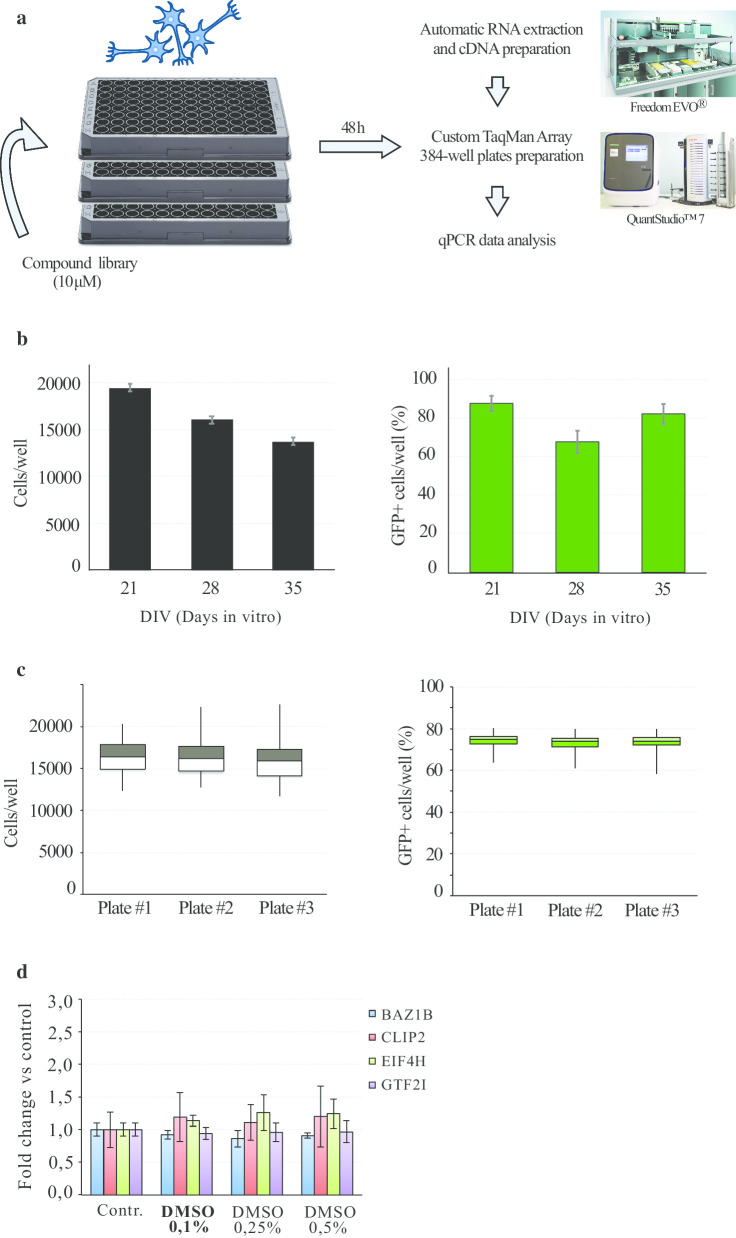
HTS workflow outline. **a** Compounds were tested at 10 μM for 48 h on NGN2 neurons seeded in 96-well plates. After RNA extraction and cDNA preparation, custom TaqMan Array 384-well plates were assembled through an automated TECAN Freedom EVO workstation. RT-qPCR were performed in QuantStudio™ 7 Flex Real-Time PCR System. **b** DAPI-stained (left) and GFP-positive (right) WBS01CN3 NGN2 neurons counted with Cellomics during differentiation. **c** Normalization panel for quantification of cell number (left) and GFP positive cells (right) in three different 96-well plates at DIV 28 (WBS01CN3 line). **d** Relative expression of BAZ1B, CLIP2, EIF4H, and GTF2I mRNA (mean ± SE) in day 28 WBS01CN3 neurons was measured by RT-qPCR, upon treatment with different DMSO concentrations. Highlighted in bold the DMSO concentration chosen for the screening

In order to screen large chemical libraries, we defined disease-relevant models for WBS and 7Dup suitable for HTS. We tested the differentiation protocol with a WBS line (WBS01CN3) to establish the proper conditions for adaptation to a miniaturized HTS format (Fig. 3a). We obtained optimal cell-plating settings for 96-well plates using poly-d-lysine coated plates by seeding 20.000 cells/well. Cellular growth and percentage of GFP positive cells were monitored by automated cell counting after Dapi-nuclear staining through 5 weeks of differentiation. An expected slight decrease of total cell number was observed over the course of differentiation, while GFP positive cells percentage remained stable around a 70%. On these bases, 28 DIV (days in vitro) has been chosen as time point to perform the in vitro assay (Fig. 3b). Good consistency and reproducibility have been found once assessed cell plating consistency for three plates from a single round of differentiation considering both cell number (Fig. 3c, left panel) and GFP positivity (Fig. 3c, right panel). Optimal morphology and proliferation characteristics of iPSC lines were checked periodically, and the lines were kept in culture for no more than 2 months, the duration of the screening.

To develop an iN-based HTS assay, considering the relevance that could have for both WBS and 7Dup in correcting the genetic imbalance, we envisaged that promising compounds could restore mRNA levels of four genes in the WBS region, namely *GTF2I*, *BAZ1B*, *CLIP2* and *EIF4H*. Therefore, we selected TaqMan qRT-PCR assays for measuring the expression levels of these genes against our internal controls *GAPDH*, *SRSF9* and *RPS18*. Transcript levels were measured after cell lysis, RNA extraction, qRT-PCR and data quantification. DMSO had no major impact on growth of iNs and on mRNA levels up to 0.5% DMSO (v/v) (Fig. 3d). Finally, having checked all the parameters, we proceeded with a moderate-sized screening of around 100 96-well plates of iNs.

**Fig. 4:**
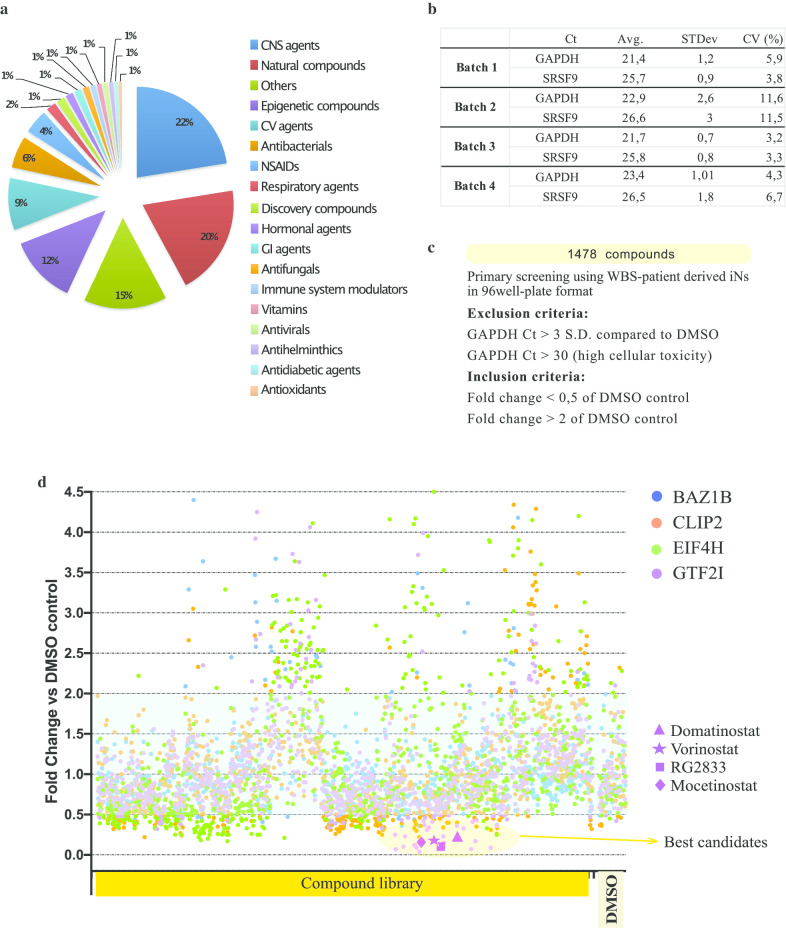
Primary screening of a pharmaceutical compound library. **a** Composition of the compound library (1478 compounds). **b** Robustness of primary HTS setup. For each batch of plates, control run statistics with average Ct values (Avg.) of GAPDH and SRSF9 housekeeping genes, their SD and CV are summarized. **c** Exclusion and inclusion criteria of the primary screening.  **d** Scatter plot of the primary screening. All compounds were tested at 10 μM for 48 h. Fold changes compared with DMSO control were plotted for each gene (BAZ1B, CLIP2, EIF4H, GTF2I) in WBS01CN3 NGN2 neurons. Selected hits are shown for GTF2I

We screened a library of 1478 small molecules in biological triplicate (4434 treatment conditions in total). Our screening library comprises an extensive variety of compounds, including e.g. central nervous system (CNS) agents, natural compounds, hormonal agents, epigenetic and immune system modulators, antioxidants (Fig. 4a). The compounds were selected analyzing an internally available Chemical Collection of more than 200.000 compounds composed by FDA-approved drugs, bioactive compounds (including preclinical and clinical compounds), a kinase target library, a fragment library and a commercially available screening library. During the process of selection, pain assay interference compounds and molecules presenting known reactive and/or toxic moieties have been filtered and removed. Among the remaining compounds, approved drugs, preclinical and clinical molecules have been preferred with the aim of accelerating the path from discovery to patients.

Epigenetic compounds have been largely represented inside the preclinical compounds envisaging a relevant role of chromatin perturbation and epigenetic modifications for the considered pathology.

Compounds were screened at 10 μM in 0.1% DMSO, with each plate containing three DMSO control wells. We used the WBS patient-derived monoclonal line WBS01CN3 for the first HTS, looking for molecules that restore the gene dosage of *BAZ1B*, *CLIP2*, *EIF4H* and *GTF2I* in patient-specific iNs. To validate our qRT-PCR assay, we measured parameters of the screening workflow, as coefficient of variation (CV), to demonstrate consistency in Ct values in the four batches of cell plates. Differences in Ct values were minimal among replicate wells of the same batch giving a CV < 20% (Fig. 4b). The strategy we used to nominate candidate hits out of the 1478 compounds tested in triplicate was based on fold-difference analysis of WBS genes to housekeeping genes, comparing compound wells to DMSO control-treated wells. Compounds that gave rise to increased GAPDH Ct values > 3 SD, compared to DMSO values, or to GAPDH Ct values > 30 were excluded from the analysis, as revealing high cellular toxicity (Fig. 4c).

Hits were defined as more than twofold increase or less than 0.5-fold decrease in at least one out of the four genes (Fig. 4c, d). Thirty-five compounds fulfilled the first criteria and were further analyzed under standard 6-well plate culture conditions, but we did not confirm any compound able to increase more than twofold the expression levels of the target genes in WBS neurons.

### HDAC inhibitors specifically lower the mRNA and the protein levels of GTF2I in 7Dup induced neurons

**Fig. 5:**
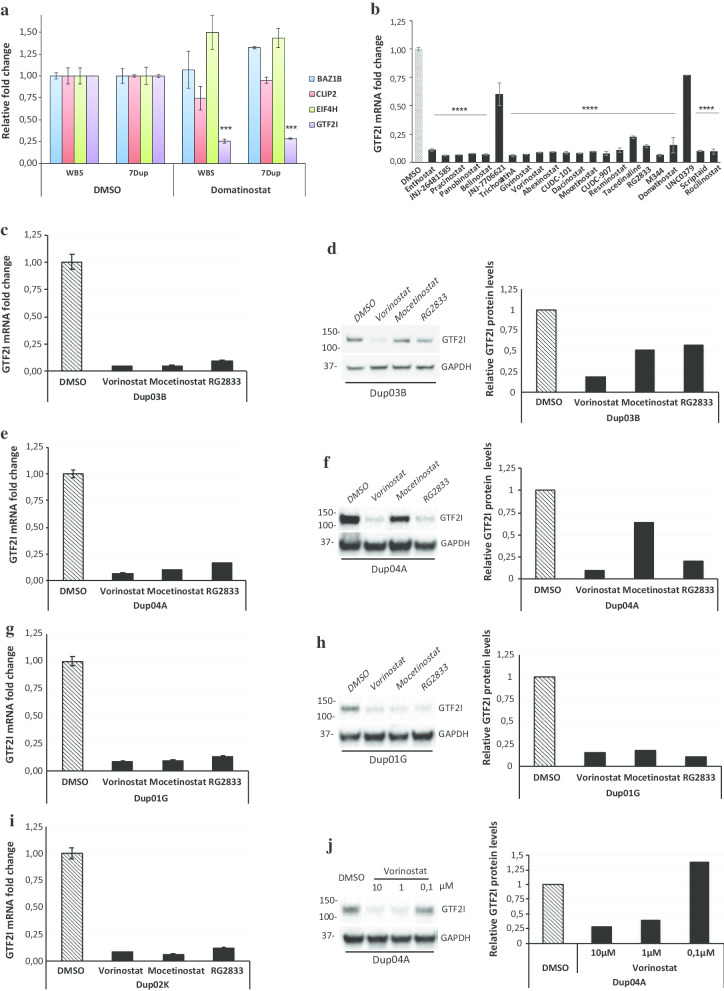
HDAC inhibitors lower the mRNA and the protein levels of GTF2I in 7Dup iNs. **a** Relative expression of BAZ1B, CLIP2, EIF4H, and GTF2I mRNA (mean ± SD) in WBS01CN3 and Dup02K iNs (*n* = 2) treated with Domatinostat 10 μM compared to control (DMSO). Error bars represent variation between lines of the two genotypes (Holm-Sidak-corrected *t* test ***P < 0.001). **b** Relative expression of GTF2I mRNA (mean ± SD) in two 7Dup-derived iNs, Dup02K and Dup01G, treated with different classes of epigenetic compounds compared to control. Error bars represent variation between the two above-mentioned iN lines (one way ANOVA test: *P < 0.05, **P < 0.005, ***P < 0.0005, ****P < 0.0001). Relative expression of GTF2I mRNA (mean ± SE) in Dup03B (**c**), Dup04A (**e**), Dup01G (**g**) and Dup02K (**i**) iNs treated with vorinostat 10 μM, mocetinostat 10 μM and RG2833 10 μM compared to control. Relative expression was measured by RT-qPCR, normalized against GAPDH-SRSF9-RPS18 geometric mean. In **c**, **e**, **g**, **i** error bars represent variation between three technical replicates. Protein levels of GTF2I in Dup03B (**d**), Dup04A (**f**) and Dup01G (**h**) iNs treated with vorinostat, mocetinostat and RG2833 10 μM each compared to control. Immunoblot (left) and densitometric analysis (right). **j** Protein levels of GTF2I in Dup04A iNs treated with different concentrations of vorinostat (0,1-1-10 μM) compared to control. Immunoblot (left) and densitometric analysis (right)

While the above results uncover the WBS gene dosage as particularly resilient to any attempt at positive transcriptional modulation, at least within the chemical universe we explored in this screening, we noticed negative transcriptional modulation by specific compounds such as domatinostat, which decreased the expression levels of *GTF2I*, in the WBS genetic background (Fig. 4d), without any significant transcriptional modulation of the other three genes *BAZ1B, CLIP2* and *EIF4H* compared to the vehicle control (DMSO) (Fig. 5a). We thus reasoned that such compounds that are able to further lower, even in a haploinsufficient context, critical WBSCR genes such as *GTF2I* could prove particularly useful to rescue the transcriptional imbalance of the symmetrical 7q11.23 syndrome. For this purpose, we generated several polyclonal lines, i.e. Dup03B, Dup04A, Dup01G and Dup02K, using the ePB based system containing the same NGN2 cassette that we used for the monoclonal lines. We thus tested domatinostat at the same concentration (10 μM for 48 h) in 28-day-old 7Dup iNs (i.e., harboring the symmetrically opposite genetic lesion) and confirmed the specific effect of lowering *GTF2I* levels (Fig. 5a). We thus set out to expand this observation to other compounds within the epigenetic subset of our HTS library. We observed that 20 out of 22 epigenetic compounds tested in the validation process, lowered *GTF2I* levels in 7Dup iNs, and, interestingly, they all belong to the class of HDAC inhibitors (Fig. 5b). Indeed, both JNJ-7706621 and UNC0379, which are, respectively, a CDK inhibitor and a histone methyltransferase inhibitor, have no effect on *GTF2I* mRNA (Fig. 5b).

In order to characterize these hits in greater detail and prioritize them, we carried out an analysis of their selectivity profile as well as of the chemical diversity and of their pharmacokinetic properties. This led us to select the following three compounds, vorinostat, mocetinostat and RG2833, according to the parameters of (i) blood–brain barrier (BBB) penetration ability, (ii) FDA approval, and (iii) HDAC class/type selectivity (Table 1). On this basis, we went on to validate the *GTF2I*-lowering effect of the three selected compounds on multiple 7Dup patient-derived lines, so as to secure the generalizability of our findings across a heterogeneity of human backgrounds harboring the 7q11.23 duplication. We confirmed that the three selected HDACi lower the expression levels of *GTF2I* in 7Dup 28-day-old neurons derived from four genetically different iPSC lines, i.e. Dup03B (Fig. 5c), Dup04A (Fig. 5e), Dup01G (Fig. 5g) and Dup02K (Fig. 5i). The effect of HDACi on GTF2I was confirmed also at a protein level in Dup03B (Fig. 5d), Dup04A (Fig. 5f) and Dup01G (Fig. 5h) iNs. Specifically, vorinostat emerged as the most promising in reducing consistently also the protein levels of GTF2I, to a degree comparable to the transcriptional readout and in a reproducible manner across different patient-derived iNs, while mocetinostat and RG2833 showed more variable correspondence between transcript and protein level assays across patient-derived iNs.

**Table 1:** Selection criteria for HDACi

Molecule name	Selectivity	Development status	Maximum development phase	FDA approved	BBB penetration	Half-life (h)
Entinostat	HDAC 1, 3	Investigational	3	0	Limited/poor	33–150 [63]
JNJ-26481585	Pan HDAC	Investigational	2	0	Not reported	8,8 [64]
Pracinostat	Pan HDAC	Investigational	2	0	Yes in mice	5,6–8,9 [65]
Panobinostat	Pan HDAC	Marketed	4	1	Limited	16 [66]
Belinostat	Pan HDAC	Marketed	4	1	Limited	1,5 [67]
Trichostatin A	Pan HDAC	Investigational	1	0	Limited	Not determined
Givinostat	Pan HDAC	Investigational	3	0	Yes	6,9 [68]
Vorinostat	Pan HDAC	Marketed	4	1	Yes	1–2 [69]
Abexinostat	Pan HDAC	Investigational	1	0	Yes	4 [70]
CUDC-101	Pan HDAC, Her, EGFR	Investigational	1	0	Not reported	4,4 [71]
Dacinostat	Pan HDAC	Investigational	2	0	Not reported	6–15 [72]
Mocetinostat	Class I selective	Investigational	2	0	Yes in mice	7–11 [73]
CUDC-907	Pan HDAC, PI3K	Investigational	2	0	< 10% in mice	3 [74]
Resminostat	HDAC 1, 3, 6	Investigational	2	0	Not reported	3 [75]
Tacedinaline	Class I selective	Investigational	3	0	15–45% in monkey	8,5–10 [76]
RG2833	HDAC 1, 3	Investigational	1	0	Yes in mice	6–10 [77]
M344	Pan HDAC	Discovery		0	Yes in mice	Not determined
Domatinostat	Pan HDAC, LSD1	Investigational	1	0	Not reported	20 [78]
Scriptaid	Pan HDAC	Discovery		0	Not reported	Not determined
Rocilinostat	HDAC 6	Investigational	2	0	Not reported	3 [79]

In order to define the compounds’ effect across the 7q11.23 interval, we expanded our gene expression analysis testing the effect of vorinostat, mocetinostat, and RG2833 on 13 additional genes of the WBS region, prioritizing those most relevant to the neuronal pathophysiology of the 7Dup (Additional file 3: Fig. S2A) [23]. Interestingly, we observed that the three compounds decrease the expression levels of *GTF2IRD1* and *VPS37D*, along with *GTF2I* (Additional file 3: Fig. S2B, right panel), while vorinostat and mocetinostat slightly decrease the expression level also of *CLIP2*. Four genes show a trend of increase upon treatment while most of the remaining genes in the region show no major changes in expression levels (Additional file 3: Fig. S2B, left and central panel) (Additional file 4).

Given the key role played by GTF2I in the pathophysiology of both syndromes [19–21, 33–35], alongside initial evidence linking its polymorphisms to sociability metrics in the wider population [36], the largely selective effect of the three compounds provides thus a promising basis for the translation of these findings, especially in the context of a chronic treatment meant to provide cognitive/behavioral amelioration (GTF2I dosage-dependent) while leaving largely unaffected the regulation of other 7q11.23 genes with pleiotropic functions.

Combining these observations, we thus selected vorinostat as our top lead, and treated Dup04A neurons with different concentrations of vorinostat to define a dose–response range, finding that the reduction of GTF2I protein levels was maintained down to 1 μM (Fig. 5j).

## Discussion

WBS and 7Dup are two paradigmatic neurodevelopmental disorders whose unique alignment of symmetrically opposite CNV and symmetrically opposite phenotypes in sociality and language provides unique glimpses into the molecular architecture of ASD. We previously characterized the effect of 7q11.23 CNV in early human lineages through the first and largest cohort of iPSC for a disease-causing symmetrical CNV. This revealed major transcriptional dysregulation already apparent at the pluripotent state and that was further exacerbated upon differentiation in disease-relevant lineages, including cortical neural progenitors [22]. Subsequent work by us and others has meanwhile expanded the characterization of 7q11.23 iPSC-based disease models to the morphofunctional level, respectively, in neural crest lineages harboring the symmetrically opposite 7q11.23 dosage [25] and in neuronal lineages carrying the 7q11.23 hemydeletion [32].

Here we present the first exploration, via HTS, of a large chemical space in search of clinically relevant compounds to restore the transcriptional dosage of key WBSCR genes, that led us to the following results.

First, we introduced an adaptation of the NGN2-driven conversion of iPSCs into functional iNs [29, 37] to an automation-intensive HTS format, which can serve as template to streamline further drug screening and/or repurposing campaigns targeting cortical glutamatergic neurons. Specifically, this entailed benchmarking of HTS-proof conditions attuned to the specific challenges of patient-derived iPSCs and iNs, including comparison of culture conditions or modes of NGN2 transgene insertion (exposing the value of the monoclonal line used in the primary HTS campaign to minimize confounding variables, followed by validation in polyclonal lines derived from multiple patients through the easily scalable polyclonal format).

Second, we identified HDAC inhibition as a powerful and surprisingly specific chromatin intervention for rescuing the aberrant transcriptional levels of *GTF2I*, the cardinal gene involved in by 7q11.23 CNV. HDACi prevent the deacetylation of histones thereby facilitating gene expression. Intensively studied for treatments of different malignancies, from hematological entities to solid tumors [38, 39], HDACi have also been probed in models of neurodegenerative disorders, such as Alzheimer's [40, 41], Parkinson’s [42], Huntington's diseases [43], and diabetic neuropathic pain [44]. Indeed, although previous studies highlighted a link between HDAC inhibition and improvement of social cognition in different mouse models of ASD [17, 45, 46], and functional recovery in cortical neurons in *MECP2* duplication syndrome [47], the use of HDACi in 7Dup patients has never been anticipated. Here we identified and confirmed three HDACi (vorinostat, mocetinostat and RG2833) that are able to reduce GTF2I expression both at a transcription and at the protein level in 7Dup iNs. In particular, vorinostat is an FDA-approved Pan HDAC inhibitor that crosses the BBB [48]; mocetinostat is a class I selective HDACi that passes the BBB in mice [49], and RG2833 is a brain-penetrant HDACi with a specificity for HDAC1 and HDAC3 [50] (Table 1). The common characteristic of these compounds, which grounded our rational for selecting them for validation amongst the other HDACs leads emerged from the HTS, is the ability to pass the BBB, an obviously crucial aspect for neurodevelopmental disorders. Importantly, at present vorinostat is among four HDACi, along with panobinostat, belinostat and depsipeptide (romidepsin), that have already received FDA approval for the treatment of a number of conditions, including refractory cutaneous T cell lymphoma, refractory multiple myeloma and peripheral T cell lymphoma, respectively [51–54]. Besides existing approval, our results provide additional support for vorinostat as the most promising HDACi amongst the ones we identified. Specifically, we probed the effect of the three compounds also at the protein level, aiming at scoring the best performance on two criteria: (1) the narrow range of the effect, i.e. privileging the compound best capable of fine-tuning the level of GTF2I, thus avoiding an excessive decrease that might spill into the WBS dosage range; and (2) the robustness of this fine-tuned effect across different patients. On this basis, we observed that the mild effect observed with mocetinostat and RG2833 at a protein level appears to be patient-dependent, whereas vorinostat emerges clearly as the most reliable in reducing the protein levels of GTF2I in iNs derived from three different patients. Finally, its effect is maintained down to 1 μM, the dose corresponding to the clinically active tolerated relevant concentration approved for oncology indications [55].

Third, while the effect of HDACi on GTF2I is very specific with respect to the other three genes we had scored as targets in our screening, it is arguably indirect. This is consistent with the observation, as summarized in Table 1, that the most represented HDACi specificities among the compounds we identified are for different classes of HDAC: HDAC 1, 3, and 6. HDAC 1 and 3 are included in class I HDAC, while HDAC 6 belongs to another class (IIb). Specifically, HDAC 1 is expressed primarily in neurons and it mainly functions in combination with HDAC2 in several repressor complexes; HDAC3 is the most highly expressed class I HDAC in the brain and it is also predominantly expressed in neurons, playing an essential role in brain development [56]; lastly, HDAC6 is involved in processes related to neurodegeneration, binding to ubiquitinated protein aggregates [57].

This diversity of pathways whose inhibition converges on GTF2I is not surprising given the observations from several studies demonstrating how HDAC inhibitors can cause both up- and downregulation of gene expression patterns [58–61], pointing to the fact that HDAC inhibitions also alter the expression of additional enzymes or co-factors which in turn will act as activators or repressors of other downstream genes.

Finally, the specificity of effect on GTF2I underscores the possibility that even in disorders caused by fairly large CNV encompassing multiple genes, it is possible to identify compounds that, albeit acting through major regulatory pathways such as histone deacetylation, end up exerting, in the context of patient-derived disease-relevant cell types, an exquisitely specific effect. For clinical translation this is potentially highly relevant, since in multi-gene CNV disorders for which one gene is particularly critical (as the case of *GTF2I* for 7q11.23 CNV), selective therapies may likely have fewer side effects than those modulating the expression of the entire CNV.

Together, our results establish the power of ASD patient-specific neurons for drug discovery and/or repositioning through HTS and identify HDACi, and especially vorinostat, as particularly promising repurposed compounds for 7Dup, whose effects warrant further characterization in complementary pre-clinical models such as patient-derived cortical brain organoids, that we characterized recently for their highly reproducible attainment of corticogenesis milestones [62], and *GTF2I* CNV murine models [20], that recapitulate in vivo some of the salient ASD phenotypes relevant to 7Dup.

## Limitations

Insofar as beyond the scope of the current work, in this study we did not address the molecular mechanisms whereby HDAC inhibitors act on *GTF2I*. Consistent with the aim of a HTS campaign, the identification of lead compounds has been the focus of this study. The lead compounds will now be advanced to further testing in additional models, including patient-derived brain organoids and mouse models recapitulating the gene imbalances of the 7q11.23 microduplication, in order to validate their efficacy in rescuing phenotypes across multiple functional layers within a translational pipeline toward clinical use. This will include a full characterization of the synaptic physiopathology of the two conditions.

## Conclusions

Drug repositioning has the potential to provide new therapeutic alternatives for patients as well as “new” innovative use for “old” drugs thus delivering relevant clinical improvement while reducing their clinical development time compared to de novo development of new chemical entities.

Considered the unmet medical need in the ASD field, our HTS-derived results represent a unique opportunity to develop first-in-class therapeutic agents for the 7Dup syndrome and possibly other neurodevelopmental conditions and an intriguing prospect to investigate the link between HDAC inhibition and GTF2I regulation. Finally, effective treatments of 7Dup core symptoms will also help to reduce the staggering physical and mental stress on patients’ caregivers, along with the financial burden involved in managing this disease, conferring a great benefit to the society.

## Supplementary information

**Additional file 1: Table S1**. Quality control of iPSC lines.

**Additional file 2: Figure S1**. Neuronal marker expression and Sholl analysis in NGN2 neurons.

**Additional file 3: Figure S2**. Effect of HDAC inhibitors on the expression levels of WBSCR genes.

**Additional file 4.** Supplemental figure legends and experimental procedures. Virus preparation for Sholl analysis; Neuronal infection; Image acquisition; Morphological analysis.

## Data Availability

All data generated or analyzed during this study are included in this article and its supplementary information files.

## Abbreviations

ADHD: Attention deficit hyperactivity disorder
ASD: Autism spectrum disorder
BBB: Blood–brain barrier
CNS: Central nervous system
CNV: Copy number variation
CV: Coefficient of variation
DIV: Days in vitro
7Dup: 7q11.23 microduplication syndrome
ePB: Enhanced PiggyBac
FDA: Food and Drug Administration
GTF2I: General transcription factor II-I
HDACi: Histone deacetylase inhibitors
HTS: High-throughput screening
iNs: Induced neurons
ID: Intellectual disability
iPSCs: Induced pluripotent stem cells
LSD1: Lysine demethylase 1
NDD: Neurodevelopmental disorders
NGN2: Neurogenin-2
rtTA: Reverse tetracycline transactivator
WBS: Williams-Beuren syndrome
WBSCR: WBS critical region
