# Supplementary figures and images for: High-throughput screening identifies histone deacetylase inhibitors that modulate GTF2I expression in 7q11.23 microduplication autism spectrum disorder patient-derived cortical neurons
Source: Mol Autism. 2020 Nov 19;11:88. doi: 10.1186/s13229-020-00387-6 (PMC7677843; doi:10.1186/s13229-020-00387-6)

**A**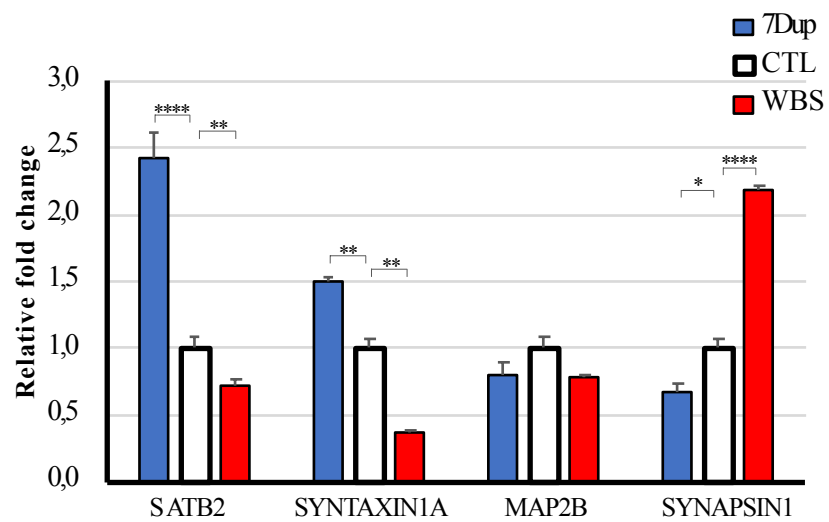**B**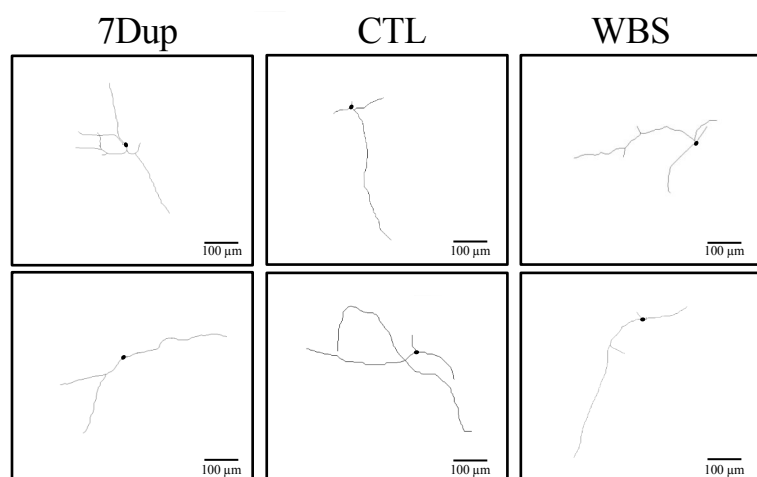**C**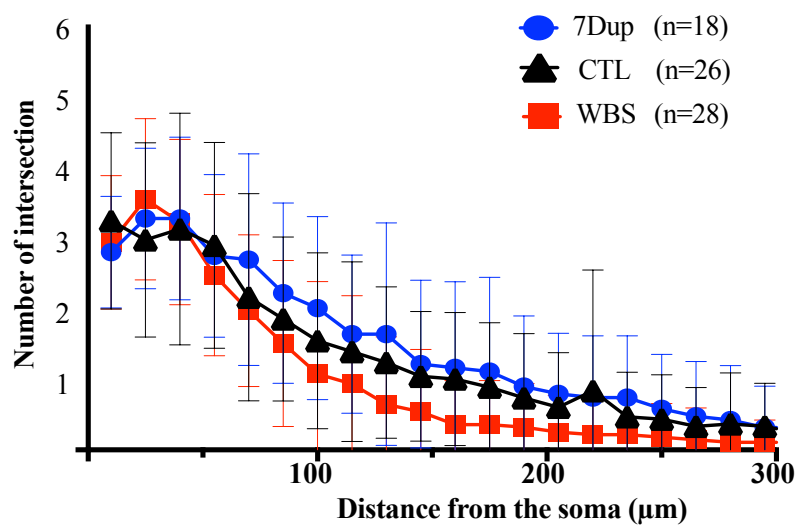

Supplement: Supplementary file 2 — Additional file 2: Figure S1. Neuronal marker expression and Sholl analysis in NGN2 neurons. [file 13229_2020_387_MOESM2_ESM.pdf]

A

Chr.7

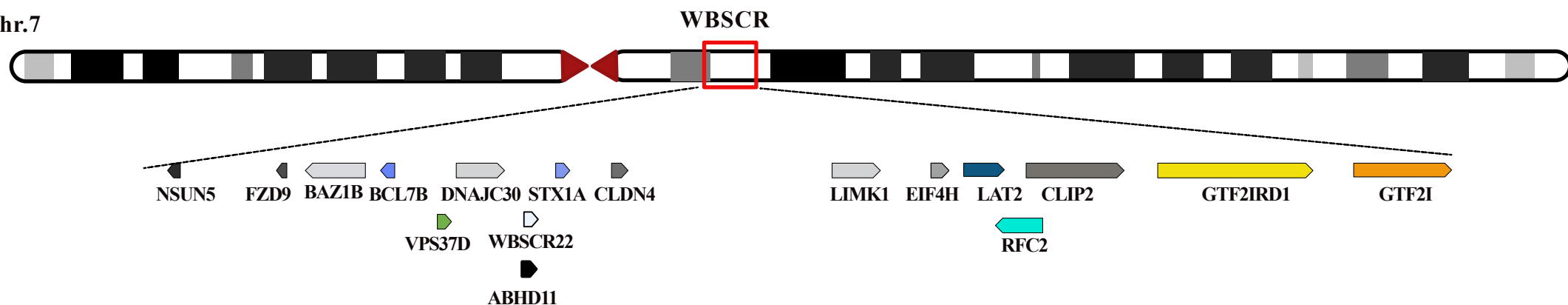

B

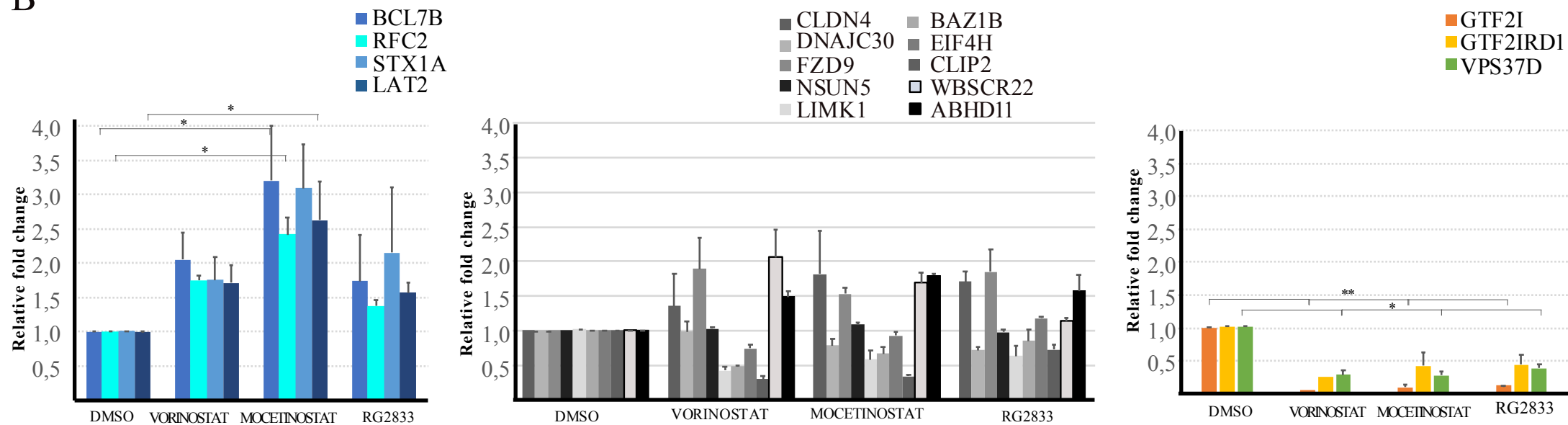

Supplement: Supplementary file 3 — Additional file 3: Figure S2. Effect of HDAC inhibitors on the expression levels of WBSCR genes. [file 13229_2020_387_MOESM3_ESM.pdf]
