# Supplementary material for: High-throughput screening identifies histone deacetylase inhibitors that modulate GTF2I expression in 7q11.23 microduplication autism spectrum disorder patient-derived cortical neurons
Source: Mol Autism. 2020 Nov 19;11:88. doi: 10.1186/s13229-020-00387-6 (PMC7677843; doi:10.1186/s13229-020-00387-6)
Supplement: Supplementary file 4 — Additional file 4. Supplemental figure legends and experimental procedures. Virus preparation for Sholl analysis; Neuronal infection; Image acquisition; Morphological analysis. [file 13229_2020_387_MOESM4_ESM.docx]

**SUPPLEMENTAL FIGURE LEGENDS**

**Fig. S1 Neuronal marker expression and Sholl analysis in NGN2 neurons. A**  RT-qPCR analysis of neural markers by differentiated neurons according to the genotype. Relative expression of SATB2, Syntaxin 1A, MAP2, and Synapsin 1 to TBP levels. Results were arbitrarily normalized to mRNA levels of CTL neurons. (mean ± S.E., n=3, asterisks indicate statistical significance according to a one way ANOVA test: **P* < 0.05, ***P* < 0.005, ****P* < 0.0005, *****P* < 0.0001). CTL: Ctl01C, Ctl08A; WBS: WBS01CN3, WBS02C; 7Dup: DUP01GN4, Dup02K. **B** Representative images of tracings from 7Dup, healthy CTL and WBS iPSC-derived neurons. **C** Sholl analysis of dendrites from WBS, 7Dup and CTL neurons revealed no significant alterations in dendritic morphology (mean ± S.D., Two-way ANOVA test shows no statistical significant differences). Lines used CTL: Ctl01C; WBS: WBS01C, WBS02C; 7Dup: Dup01G, Dup03B.

**Fig. S2 Effect of HDAC inhibitors on the expression levels of WBSCR genes. A** Schematic representation of WBSCR genes analyzed after treatment of 7Dup iNs with the three HDACi indicated below. **B** Relative expression levels of 17 genes in WBSCR in Dup03B and Dup04A iNs treated with 10μM Vorinostat, Mocetinostat and RG2833 compared to DMSO controls (mean ± S.D. asterisks indicate statistical significance according to a one way ANOVA test: **P* < 0.05, ***P* < 0.005).

**SUPPLEMENTAL EXPERIMENTAL PROCEDURES**

**Virus preparation for Sholl analysis.** Five million HEK 293T cells were plated in 10 cm plates and grown in 10% fetal bovine serum in DMEM. On the next day, cells were transfected with plasmids for gag-pol (10 μg), rev (10 μg), VSV-G (5 μg) and the target construct (15 μg) CaMKIIα-mKO2, using the calcium phosphate method [79]. On the next day, the medium was changed. On the day after, the medium was spun down in a high-speed centrifuge at 30,000g, at 4 °C for 2 h. The supernatant was discarded and 100 μl of PBS were added to the pellet and left overnight at 4 °C. On the next day, the solution was triturated, distributed into 10-μl aliquots and frozen at −80 °C.

**Neuronal infection.** At day 4 of the differentiation protocol, neurons were infected with virus bearing CaMKIIα-mKO2; specifically, 10 μl virus per 6cm plate from a standard preparation (see Virus preparation). At this time, an appropriate number of vials of mouse astrocytes were thawed into a 10 cm plate, in order to obtain at least 1.25 million of astrocytes at day 8. At day 8, infected neurons were digested with accutase for 5 min., washed with PBS, counted, and seeded at a total density of at least 30.000 cells/cm^2^ (300.000 cells/well in a 6-well plate) in a 1:50 ratio with not infected neurons and in a 1:1 ratio with mouse astrocytes, in poly-D-lysine-coated coverslips. Over the following weeks, the coverslips were monitored and those with at least 10 visible individual neurons were kept for image acquisition.

**Image acquisition.** For morphometric analysis, images of neurons were acquired at 10x magnification using the Leica DM6 Multifluo Fluorescence Microscope. Image acquisition was done in a semi-automated manner, with manual picking of individual neurons and batch acquisition. Two channels were acquired per batch for GFP and mKO2.

**Morphological analysis.** All analyses were completed in Fiji (ImageJ). Dendrite length was characterized using the Simple Neurite Tracer ImageJ plugin. For Sholl analysis, the center of concentric spheres was defined as the center of the soma, and a 10 μm radius interval was used. In order to compare the Sholl analysis curves between genotypes, a two-way ANOVA test was performed. P-values < 0.05 were considered statistically significant.

**Bibliography related to experimental procedures**

79. Zaslavsky K, Zhang W-B, McCready FP, Rodrigues DC, Deneault E, Loo C, et al. SHANK2 mutations associated with autism spectrum disorder cause hyperconnectivity of human neurons. Nat Neurosci. 2019 Mar 25;22(4):556–64.
